# A mediation analysis of the role of total free fatty acids on pertinence of gut microbiota composition and cognitive function in late life depression

**DOI:** 10.1186/s12944-024-02056-6

**Published:** 2024-02-29

**Authors:** Yan Chen, Jiarong Li, Dansheng Le, Yuhan Zhang, Zhengluan Liao

**Affiliations:** 1 Center for Rehabilitation Medicine, Department of Psychiatry, Zhejiang Provincial People’s Hospital (Affiliated People’s Hospital), Hangzhou Medical College, 158 Shangtang Rd, Hangzhou, 310014 Zhejiang People’s Republic of China; 2 Institute of Immunology and College of Veterinary Medicine, Nanjing Agricultural University, 1 Weigang, Nanjing, 210095 Jiangsu Province China; 3 The Second Clinical College of Zhejiang, Chinese Medical University, Hangzhou, 310053 Zhejiang China

**Keywords:** Late-life depression, Gut microbiota, Lipid metabolism, Mediation analysis

## Abstract

**Background:**

Extensive evidence demonstrates correlations among gut microbiota, lipid metabolism and cognitive function. However, there is still a lack of researches in the field of late-life depression (LLD). This research targeted at investigating the relationship among gut microbiota, lipid metabolism indexes, such as total free fatty acids (FFAs), and cognitive functions in LLD.

**Methods:**

Twenty-nine LLD patients from the Cognitive Outcome Cohort Study of Depression in Elderly were included. Cognitive functions were estimated through the Chinese version of Montreal Cognitive Assessment (MoCA). Blood samples were collected to evaluate serum lipid metabolism parameters. Fecal samples were evaluated for gut microbiota determination via 16S rRNA sequencing. Spearman correlation, linear regression and mediation analysis were utilized to explore relationship among gut microbiota, lipid metabolism and cognitive function in LLD patients.

**Results:**

Spearman correlation analysis revealed significant correlations among *Akkermansia* abundance, total Free Fatty Acids (FFAs) and MoCA scores (*P* < 0.05). Multiple regression indicated *Akkermansia* and total FFAs significantly predicted MoCA scores (*P* < 0.05). Mediation analysis demonstrated that the correlation between decreased *Akkermansia* relative abundance and cognitive decline in LLD patients was partially mediated by total FFAs (Bootstrap 95%CI: 0.023–0.557), accounting for 43.0% of the relative effect.

**Conclusion:**

These findings suggested a significant relationship between cognitive functions in LLD and *Akkermansia*, as well as total FFAs. Total FFAs partially mediated the relationship between *Akkermansia* and cognitive functions. These results contributed to understanding the gut microbial-host lipid metabolism axis in the cognitive function of LLD.

## Background

Late-life depression (LLD) refers to depression that occurs over 60 years of age, and primary depression first occurring in old age is one of the special forms, which is the object of this study. Cognitive decline is a prominent symptom in LLD patients, thereby increasing the risk of dementias [1]. Authoritative findings indicated that in association with late-onset LLD (onset after age 70), the morbidity risk of Alzheimer's disease (AD) nearly doubles [2]. Current medications for the cognitive impairment of LLD often exhibited poor efficacy, which leads to the persistence of symptoms [3]. Therefore, exploring the mechanism underlying cognitive impairment in LLD and identifying new therapeutic targets is of paramount importance.

Recently, the microbiome-gut-brain axis emerged to be a concept closely associated with brain function [4] and the pathogenesis of various neuropsychiatric diseases. This axis bi-directionally regulated the intestinal microbiome and brain function through neuroanatomical pathways, neuroimmune and neuroendocrine pathways, intestinal mucosal barrier, microbial metabolites, and blood–brain barrier. Studies demonstrated gut microbiota strongly relates to cognitive function. For instance, Zhou [5] discovered that *Sphingomonas* exhibited a negative association with overall cognitive function in AD patients, whereas *Anaerobacterium* and *Papillibacter* were positively related to cognitive function. Mice transplanted with feces from patients with Schizophrenia showed impairments in learning ability, which was similar to their donors [6]. *Lactobacillus rhamnosus* could regulate cognitive function in anxious–depressed mice [7]. Numerous researches reported the abnormalities in serotonin signaling pathway could be mediated by probiotics, further confirming vital roles of gut microbiota in cognitive symptoms of depression in the elderly [8–10]. However, to date, the roles of the gut microbiome in cognitive deficits among LLD patients remain unexplored.

Most research concentrated on the impact on gut microbial metabolites on neurotransmitters and neuroinflammation, with a focus on the proximal part of the nervous system [11]. A variety of researchers claimed the nonnegligible pertinence among the intestinal flora, endocrine and metabolic pathology [12, 13], such as obesity and diabetes [4]. Additionally, studies also found that the gut microbiota played an important role in lipid metabolism [14]. The increase of specific depression-related microbiota, such as *Firmicutes* and *Streptococcus* could up-regulate the levels of cholesterol substances [15]. Academic circles emphasized the correlation between lipid metabolism and cognitive function. For example, the apolipoprotein E (ApoE) gene [16], apolipoprotein C1 (ApoC1) gene [17] and Clusterin (CLU) gene [18], which strongly related to AD pathogenesis, were involved in the regulation of lipid transport. Excessive cholesterol levels in the brain can produce more Aβ deposits, thereby increasing the risk of dementia [19]. However, few studies emphasized the impact of gut microbiota on cognitive functions in LLD through its influence on lipid metabolism.

Hence, our research targeted at exploring cognitive functions in LLD patients and their relationships with the gut microbiota composition and lipid metabolism through mediation analysis. The study hypothesized that lipid metabolism mediated the pertinence between gut microbiota and cognitive function in LLD patients.

## Methods

### Participants

All the participants or their caregivers signed informed consent forms and completed questionnaires to collect demographic information, which involved age, sex, body mass index (BMI), education years, self-reported history of hypertension and diabetes, smoking status, alcohol use history, neuropsychological assessment and gastrointestinal conditions. This research adhered the Declaration of Helsinki and obtained approval from the Ethics Committee of Zhejiang Provincial People's Hospital (2019KY184).

Untreated LLD patients were recruited from the Department of Psychiatry in Zhejiang Provincial People's Hospital from Jan. 2020 to Dec. 2021. The inclusion criteria for the LLD group were: (1) patients ranging in age from 60 to 85 years and (2) patients with first onset and met the Major Depressive Disorder(MDD) criteria in accordance with the Diagnostic and Statistical Manual of Mental Disorders, Fifth Edition (DSM-5) criteria after the age of 60 years [20].

The following patients were excluded from the study [20]: had (1) depression caused by other neuropsychiatric diseases (neurodegenerative diseases, dementia, schizophrenia, bipolar disorder, etc.); (2) serious cardiovascular, kidney, gastrointestinal, nervous system, blood system, tumor or other related physical diseases; and (3) a history of psychoactive substance abuse. In addition, on the basis of previous work, this study adopted a series of exclusion criteria to exclude other factors that may affect the gut microbiota [21, 22]: (1) had undergone gastrointestinal surgery within 5 years; (2) had current gastrointestinal diseases such as gastrointestinal bleeding, ulcers, Crohn's disease, irritable bowel syndrome, untreated *Helicobacter pylori* infection, long-term diarrhea, cancer, etc.; and (3) had used probiotics, prebiotics, antibiotics or other drugs within three months before sampling.

### Neuropsychological assessment

All patients underwent medical history assessment and psychiatric examination by two highly trained psychiatrists. On the day of enrollment, the cognitive function of LLD patients was evaluated via Montreal Cognitive Assessment (MoCA) and disease severity was evaluated via Geriatric Depression Scale 30 item (GDS-30). MoCA was designed by Professor Nasreddine in 2004 [23]. It can be used to assess cognitive domains, including attention, language, memory, executive ability, visuospatial ability, orientation, calculation and abstract thinking. The total score is 30 points, with a normal value considered to be ≥ 26 points. The GDS-30, developed by Brink et al. in 1982, serves as a depression screening scale specifically designed for the elderly [24]. The scale consists of 30 items representing the core performance of depression in the elderly, with rating categories including moderate to severe depression (above 21), mild depression (11–20), and no risk (0–10).

### Blood collection and lipid metabolism indicators measurements

After the patients were enrolled, 5 mL of peripheral venous blood was obtained on an empty stomach, and the serum was collected. Low density lipoprotein (LDL), high density lipoprotein (HDL), ApoE, apolipoprotein B (ApoB), apolipoprotein A1 (ApoA1), Triglyceride (TG), total cholesterol (TC), total FFAs, and lipoprotein A (LPPA) were assayed by AU400 automatic biochemical analyzer (Olympus Corporation, Japan).

### Gut microbiota characteristic processing

First, 5.0 g of fresh midcourse stool sample was collected 1 day before treatment and stored in a sterile container without urine. After adding a deoxyribonucleic acid stabilizer, the sample was reserved at -80℃ for examination. Next, deoxyribonucleic acid extraction was performed via nucleic acid extraction kit (Hangzhou Guhe: GHFDE100), with concentration and quality assessed using a NanoDrop luminance meter (Thermo Fisher Scientific, USA). All samples were verified through agarose gel electrophoresis. Then, the bacterial V4 fragment of 16 S rRNA was amplified utilizing the 515F (5'-GTGCCAGCMGCCGCGGTAA-3') and 806R (5'-GGACTACHVGGGTWTCTAAT-3') primers via PCR. The amplified fragments were subsequently purified with AMPure XP Beads (Beckman, USA) and assessed using Qubit dsDNA HS Kit. Later, high-throughput sequencing was conducted using the Illumina Novaseq 6000 platform with paired-END 2 × 150 bp. The data was read and filtered using intestinal flora RNA software. Similar overlapping relationships among the flora data were read and then spliced into tags. Operational taxonomic unit (OTU) analysis was conducted using VSEARCH software v2.4.4, covering de-repeating sequences (–derep_fulllength), clustering (–cluster_fast,–id 0.97), and de-chimerism (–uchime_ref) [25]. Sequences were clustered into OTUs with 97% similarity, and typical sequences of OTUs were chosen with default parameters and annotated with species via VSEARCH software with the SILVA ribosomal RNA database version 128 [26]. The OTU list was further generated, and the community composition at each taxonomic level, including species, genus, family, order, class, phylum and kingdom, was classified. OTUs below 0.001% of the total sequence in all samples were removed.

In this study, the abundance of 183 bacteria species was detected at the genus level. After reviewing the previous literature, 8 species of bacteria most likely related to cognition and emotion, *Bacteroides* [27–29]*, Prevotella* [30–32]*, Megamonas* [33–35]*, Parabacteroides* [29, 36, 37]*, Ruminococcus* [37–39], *Faecalibacterium* [33, 40, 41]*, Bifidobacterium* [36, 42, 43], and *Akkermansia* [40, 44, 45], were included for further analysis.

### Covariates

Factors known to be associated with cognitive function, gut microbiota and lipid profiles were considered as covariates, including gender, age, BMI and years of education. Self-reported and hospital episode statistics and clinical data were used to determine predepression diagnoses of hypertension and diabetes. Current smoking status (Yes/No) in self-reported data were used to classify participants into smokers and nonsmokers. Current alcohol consumption status (Yes/No) in self-reported data were used to classify participants into drinkers and nondrinkers.

### Data statistics

The continuous variables were displayed as the mean ± standard deviation (SD) or median (25th, 75th) according to whether they fit a normal distribution. Categorical variables are described by counts (%). Spearman correlation analysis was utilized for determining the relationships among MoCA score, lipid metabolism index level and intestinal microbial abundance. Since the relative abundance of *Akkermansia* did not conform to the normal distribution through Shapiro‒Wilk test (*P* < 0.001), the relative abundance of *Akkermansia* was transformed by taking the logarithm (log-transformed) [46]. The relationships among Log *Akkermansia*, FFAs and MoCA scores were analyzed by linear regression. In addition, potential confounding factors were adjusted. Finally, SPSS PROCESS Macro v3.4 (Model 4) [47] was used to present the mediation effect of FFAs in the relationship between Log *Akkermansia* and cognitive function in LLD patients. A bootstrapping of 1000 was used to estimate 95% confidence interval (CI) of the mediating effect, and to determine whether FFAs mediated the associations between Log *Akkermansia* and cognitive impairment in LLD. *P* < 0.05 (two-tailed) was considered statistically significant.

## Results

### Patient characteristics

Thirty-five LLD patients were initially enrolled. Due to the presence of diarrhea in the stool of six subjects on the day of collection, their samples were subsequently excluded from the study. Data from 29 LLD patients were obtained, with an average age of 68.83 ± 6.56. The ratio of females to males was 21:8. The score of GDS-30 was 19.86 ± 5.84, while that of MoCA was 21.55 ± 3.75 (Table 1).

**Table 1:** Demographic and clinical characteristics of patients with LLD (*N* = 29)

	LLD (*N* = 29)
Sex(F:M)	21:8
Age (year, mean ± SD)	68.83 ± 6.56
BMI (kgm^−2^, mean ± SD)	22.26 ± 2.70
Education (year, mean ± SD)	7.17 ± 4.45
Hypertension (NO. %)	6 (20.7%)
Diabetes (NO. %)	4 (13.8%)
Smoking (NO. %)	4 (13.8%)
Drinking (NO. %)	5 (17.2%)
GDS-30 (mean ± SD)	19.86 ± 5.84
Lipid metabolism	
Cholesterol (mmol/L, mean ± SD)	5.51 ± 0.88
Triglyceride (mmol/L, mean ± SD)	1.61 ± 0.67
HDL (mmol/L, mean ± SD)	1.31 ± 0.28
LDL (mmol/L, mean ± SD)	3.28 ± 0.81
ApoA-I (g/L, mean ± SD)	1.47 ± 0.27
ApoB (g/L, mean ± SD)	1.04 ± 0.27
ApoE (mg/dl, mean ± SD)	4.06 ± 1.01
Lp(a) (mg/L, mean ± SD)	416.28 ± 469.87
FFAs (μmol/L, mean ± SD)	535.76 ± 198.93
Cognition	
MoCA (mean ± SD)	21.55 ± 3.75
Visuospatial/Executive (mean ± SD)	3.07 ± 1.28
Naming (mean ± SD)	2.62 ± 0.82
Attention (mean ± SD)	5.31 ± 0.93
Language (mean ± SD)	2.31 ± 1.00
Abstraction (mean ± SD)	1.17 ± 0.71
Delayed Recall (mean ± SD)	1.72 ± 1.79
Orientation (mean ± SD)	5.10 ± 1.08
Selected microbiota	
*Bacteroides* (median (25th, 75th))	0.198(0.113,0.289)
*Prevotella* (median (25th, 75th))	0.092(0.069,0.119)
*Megamonas* (median (25th, 75th))	0.073(0.026,0.095)
*Parabacteroides* (median (25th, 75th))	0.028(0.023,0.041)
*Ruminococcus* (median (25th, 75th))	0.018(0.005,0.033)
*Faecalibacterium* (median (25th, 75th))	0.017(0.014,0.021)
*Bifidobacterium* (median (25th, 75th))	0.012(0.006,0.013)
*Akkermansia* (median (25th, 75th))	0.005(0.002,0.006)

Continuous variables were described by mean ± standard deviation or median (25th percentile, 75th percentile) according to whether they fit the normal distribution. Categorical variables are described by counting (%)

*Abbreviations*: *BMI* Body mass index, *HDRS-24* Hamilton Depression Rating Scale-24, *GDS* Geriatric Depression Scale, *HDL* High density lipoprotein, *LDL* Low Density Lipoprotein, *ApoA-I* Apolipoprotein A-I, *ApoB* Apolipoprotein B, *ApoE* Apolipoprotein E, *Lp(a)* Lipoprotein(a), *FFAs* Free fatty acids, *LLD* Late Life Depression

**Fig. 1:**
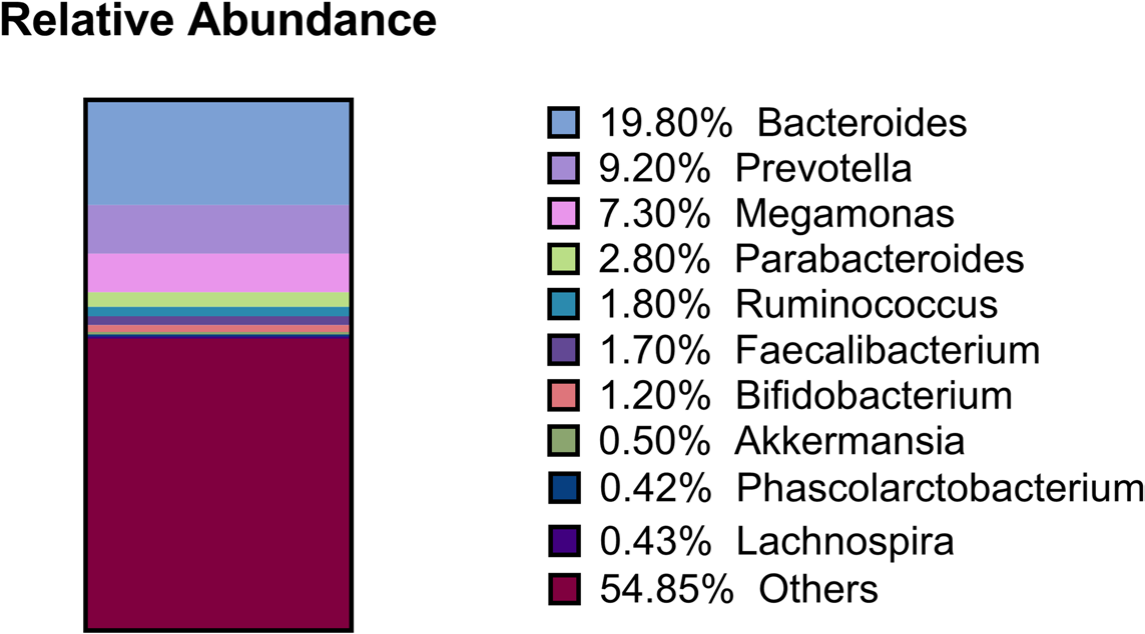
Relative abundance of gut microbiome composition in patients with LLD. Note: Relative abundance of genus-level for LLD. Abbreviation: LLD, Late-Life Depression

In this study, the abundance of 183 species of bacteria was sequenced at genus level (Fig. 1). The relative abundance of the selected eight microbial genera was as follows: *Bacteroides,* 0.198 (0.113, 0.289); *Prevotella,* 0.092 (0.069, 0.119); *Megamonas,* 0.073 (0.026, 0.095); *Parabacteroides,* 0.028 (0.023, 0.041); *Ruminococcus,* 0.018 (0.005, 0.033); *Faecalibacterium,* 0.017 (0.014, 0.021); *Bifidobacterium,* 0.012 (0.006, 0.013); and *Akkermansia,* 0.005 (0.002, 0.006) (Table 1).

The results for the lipid metabolism parameters were as follows: cholesterol, 5.51 ± 0.88; triglycerides, 1.61 ± 0.67; HDL, 1.31 ± 0.28; LDL, 3.28 ± 0.81; ApoA1, 1.47 ± 0.27; ApoB, 1.04 ± 0.27; ApoE, 4.06 ± 1.01; LPPA, 416.28 ± 469.87; and FFAs, 535.76 ± 198.93 (Table 1, Fig. 1).

### Correlations among the gut microbiota, lipid metabolism parameters and cognitive function in LLD patients

**Fig. 2:**
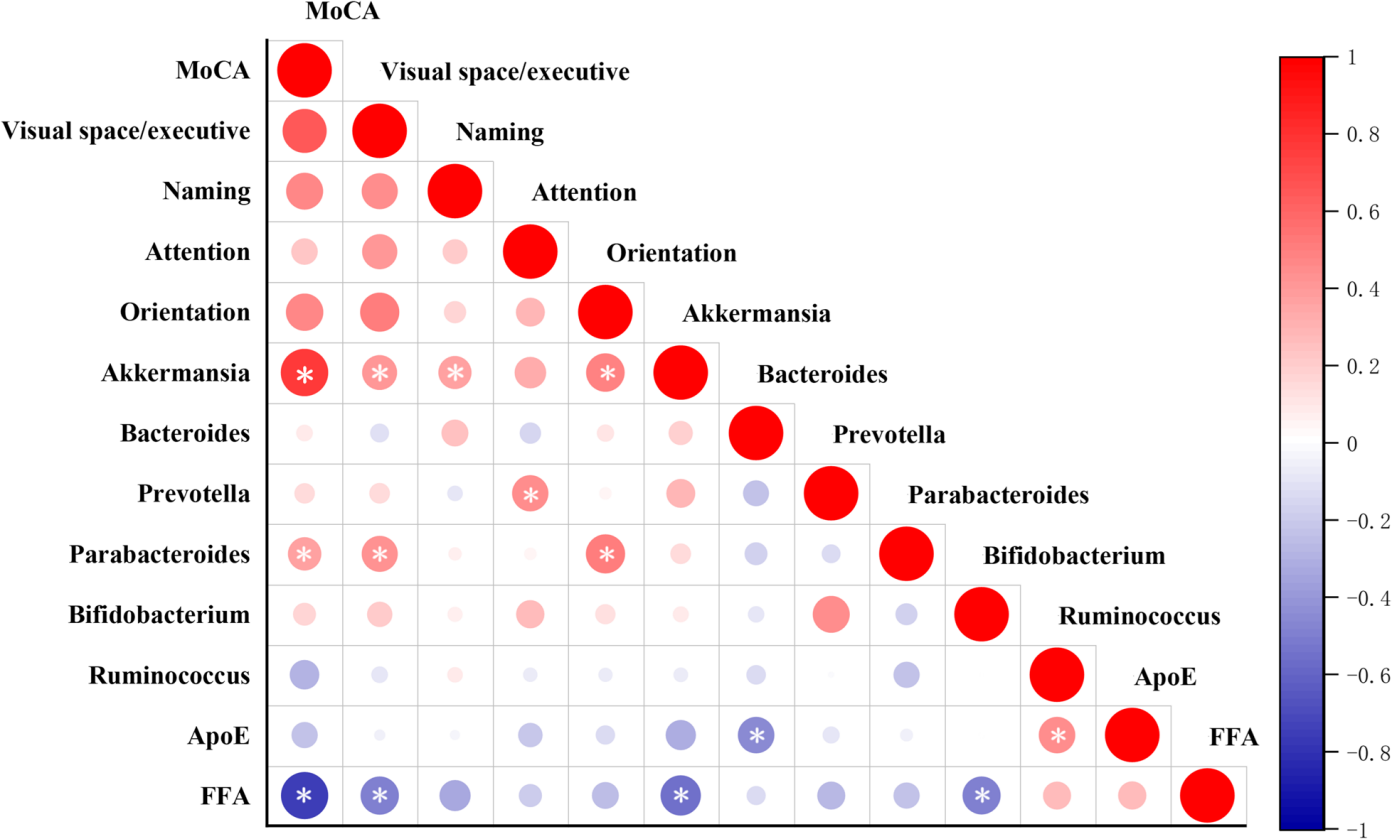
Correlations among gut microbiota, lipid metabolites and cognitive function scores. Note: Heat map revealing the relationships among gut microbes, lipid metabolites and MoCA scores in LLD. Red circle indicates positive associations, blue circle represents negative associations. The level of correlation is indicated by the degree of color. All correlations with significant differences are highlighted with asterisk (*P < 0.05). Abbreviation: MoCA, Montreal Cognitive Assessment; FFAs, free fatty acids; LLD, Late-Life Depression; ApoE, apolipoprotein E

The pertinence among gut microbiota, lipid metabolism, and cognitive function in LLD was explored by Spearman analysis. As shown in Table 2 and Fig. 2, cognitive functions were related to gut microbiota in LLD patients, as follows: MoCA total score was positively correlated with *Akkermansia* (*r* = 0.760, *P* < 0.001) and *Parabacteroides* (*r* = -0.441, *P* = 0.017). Visuospatial/executive ability positively correlated with *Akkermansia* (*r* = 0.412, *P* = 0.026) and *Parabacteroides* (*r* = 0.438, *P* = 0.018). Naming ability exhibited a positive correlation with *Akkermansia* (*r* = 0.373, *P* = 0.047), while orientation positively related to *Akkermansia* (*r* = 0.498, *P* = 0.006) and *Parabacteroides* (*r* = 0.438, *P* = 0.018). Attention showed a positive correlation with *Prevotella* (*r* = -0.441, *P* = 0.017). Secondly, total FFAs were negatively related to MoCA total scores (*r* = -0.752, *P* < 0.001) and visuospatial/executive ability (*r* = -0.492, *P* = 0.007). Thirdly, total FFAs were found to be negatively related to *Akkermansia* (*r* = -0.543, *P* = 0.002) and *Bifidobacterium* (*r* = -0.473, *P* = 0.010). ApoE showed a negative correlation with *Bacteroides* (*r* = -0.451, *P* = 0.014) and a positive correlation with *Ruminococcus* (*r* = 0.452, *P* = 0.014).

**Table 2:** Correlations among gut microbes, cognition assessment and lipid profile indexes

**Interactions between variables**		**Correlation coefficient (r)**	***p* value**
**Gut Microbiota—Cognition Assessment**			
*Akkermansia*	MoCA	0.760	< 0.001
	Visuospatial/Executive	0.412	0.026
	Naming	0.373	0.047
	Orientation	0.498	0.006
*Prevotella*	Attention	0.441	0.017
*Parabacteroides*	MoCA	0.374	0.046
	Visuospatial/Executive	0.438	0.018
	Orientation	0.517	0.004
**Lipid Metabolism- Gut Microbiota**			
Total FFAs	*Akkermansia*	-0.543	0.002
	*Bifidobacterium*	-0.473	0.010
ApoE	*Bacteroides*	-0.451	0.014
	*Ruminococcus*	0.452	0.014
**Lipid Metabolism—Cognition Assessment**			
Total FFAs	MoCA	-0.752	< 0.001
	Visuospatial/Executive	-0.492	0.007

Connections among gut microbes, cognition assessment and lipid profile indexes identified by Spearman correlation

*Abbreviations*: *MoCA* Montreal Cognitive Assessment, *FFAs* Free fatty acids, *ApoE* Apolipoprotein E

*P*value < 0.05 was displayed

### Multiple regression models for *Akkermansia*, total FFAs and the MoCA score

Uncorrected regression models revealed that both Log *Akkermansia* and total FFAs scores had significant predictive effects on MoCA scores (*P* < 0.05). Taking gender, age, years of education, hypertension, diabetes, drinking and smoking as covariates, regression analysis indicated that Log *Akkermansia* negatively predicted total FFAs content (β = -0.553, *P* < 0.004). When Log *Akkermansia* and total FFAs were separately used as predictor, Log *Akkermansia* positively predicted MoCA (β = 0.604, *P* < 0.001), total FFAs negatively predicted MoCA (β = -0.652, *P* < 0.001). When both Log *Akkermansia* and total FFAs were predictors, the effect on MoCA total score displayed remarkable significance (β = 0.343, *P* = 0.039 and β = -0.470, *P* = 0.006) (Table 3).

**Table 3:** Regression analysis between variables with LLD

Regression equation		Global fit index			Significance of regression coefficient		
**Outcome variable**	**Predictor variable**	**R**	**R**^**2**^	**F**	**β**	**t**	**P**
**Model 1**							
Total FFAs	*Akkermansia*	0.638	0.407	18.527	-0.638	-4.304	0.000
MoCA	Total FFAs	0.753	0.567	35.417	-0.753	-5.951	0.000
	*Akkermansia*	0.694	0.481	25.059	0.694	5.006	0.000
MoCA	Total FFAs	0.803	0.644	23.529	-0.524	-3.448	0.002
	*Akkermansia*				0.360	2.367	0.026
**Model 2**							
Total FFAs	*Akkermansia*	0.663	0.440	4.718	-0.553	-3.155	0.004
MoCA	Total FFAs	0.795	0.632	10.302	-0.652	-4.689	0.000
	*Akkermansia*	0.756	0.572	8.009	0.604	3.939	0.001
MoCA	Total FFAs	0.834	0.695	10.504	-0.470	-3.057	0.006
	*Akkermansia*				0.343	2.189	0.039

The relative abundance of *Akkermansia* was log-transformed as Log *Akkermansia*

Model 1: unadjusted

Model 2: Adjusted for age, sex, education, BMI, Hypertension, diabetes, smoking, drinking

β = standardized regression coefficient

*Abbreviations*: *MoCA* Montreal Cognitive Assessment, *FFAs* Free fatty acids, *LLD* Late Life Depression

### FFAs as a mediator between *Akkermansia* and cognitive functions in the LLD

**Fig. 3:**
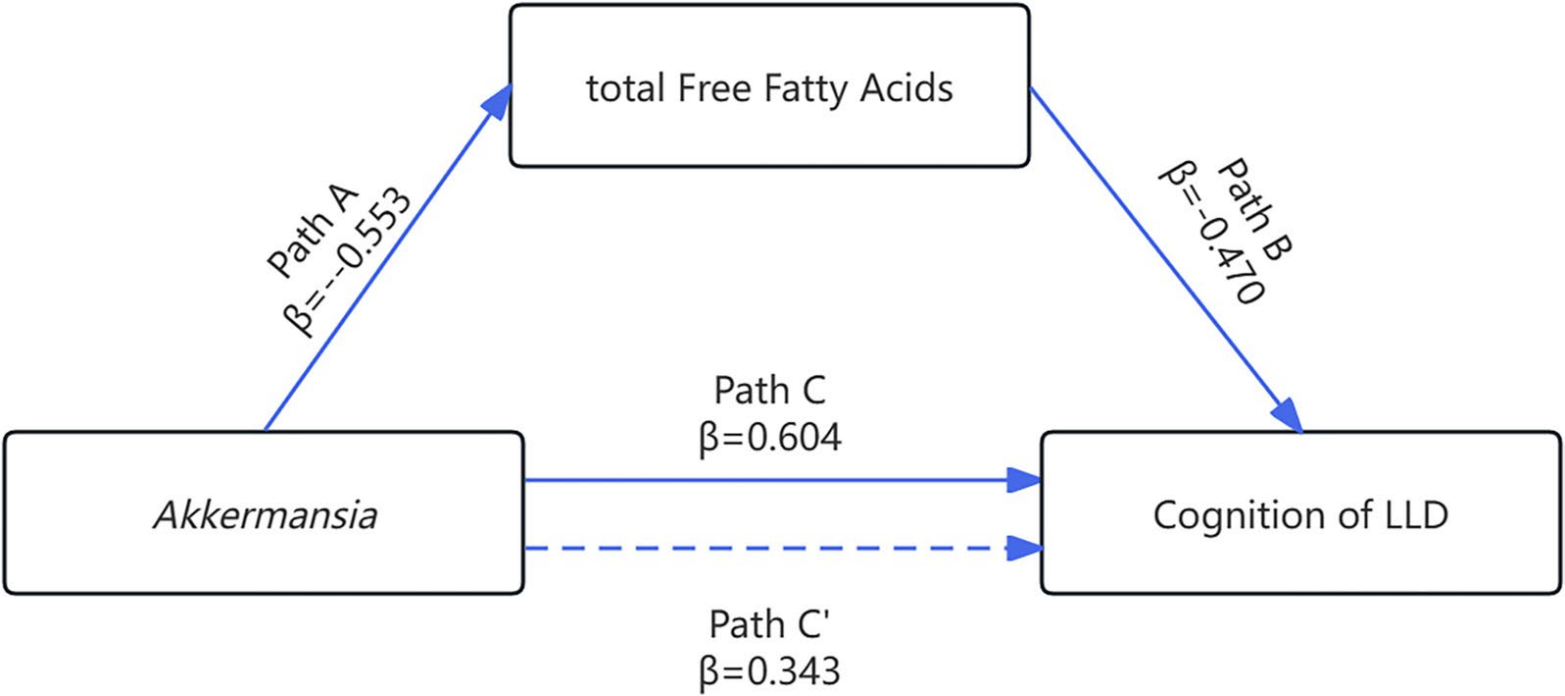
Mediation model for the role of total FFAs in the relationship between *Akkermansia* and cognitive functions. Note: Mediating models described total FFAs as a Mediator in the Relevance Between *Akkermansia* and Cognitive Function in LLD. Path A: the effect of *Akkermansia* on total FFAs; Path B: the effect of total FFAs on cognitive function; Path C: the total effect of *Akkermansia* on Cognitive Function; Path C’: the direct effect of *Akkermansia* on Cognitive Function. Abbreviation: MoCA, Montreal Cognitive Assessment; FFAs, free fatty acids; LLD, Late-Life Depression

As presented in Table 4, the direct effect of Log *Akkermansia* on MoCA was 0.343 (95%CI: 0.190–0.669). The indirect influence of Log *Akkermansia* on MoCA via total FFAs was 0.261 (95%CI: 0.023–0.557). Overall, the model demonstrated that 43.0% of the influence of Log *Akkermansia* on cognitive functions in LLD patients was mediated by total FFAs. In summary, a mediation model based on data was illustrated in Fig. 3.

**Table 4:** The mediating effect of total FFAs between *Akkermansia* and cognition in LLD

	**Effect value**	**BootSE**	**BootLLCI-BootULCI**	**Relative mediation effect**
Total effect	0.604	0.153	0.287–0.920	1
Direct effect (*Akkermansia* → MoCA)	0.343	0.157	0.190–0.669	0.570
Indirect effect (*Akkermansia* → total FFAs → MoCA)	0.261	0.135	0.023–0.557	0.430

The relative abundance of *Akkermansia* was log-transformed as Log*Akkermansia*

Adjusted for age, sex, education, BMI, Hypertension, diabetes, smoking, drinking

*Abbreviations*: *SE* Standard error, *LLCI* Lower level of confidence interval, *ULCI* Upper level of confidence interval, *MoCA* Montreal Cognitive Assessment, *FFAs* Free fatty acids, *LLD* Late Life Depression

## Discussion

In this research, cognitive functions and their correlations with gut microbiota and lipid metabolism in patients with LLD were analyzed. Correlation analysis showed that the cognitive function was positively correlated with *Akkermansia* and negatively correlated with total FFAs. Both Log*Akkermansia* and total FFAs scores exhibited significant predictive effects on MoCA scores. Moreover, total FFAs partially mediated the relationship between *Akkermansia* abundance and cognitive functions in LLD. To our best knowledge, this is the first report evaluating the relationships among cognitive function, gut microbiota and lipid metabolism in LLD patients.

This research revealed cognitive functions in patients with LLD was positively correlated with *Akkermansia*, particularly in terms of visuospatial/executive function, naming and orientation. Regression analysis indicated that *Akkermansia* served as a positive predictor of cognitive function. Previous studies found that the relative abundance of *Akkermansia* was decreased in patients suffering from Mild Cognitive Impairment (MCI) and was positively related to MoCA scores [48]. Another research assessed cognitive function in depressed mice using Morris Water Maze test and found a decrease in the relative abundance of *Akkermansia* was associated with cognitive decline [49]. Current results aligned with these findings, suggesting a decline in *Akkermansia* related to cognitive impairment in LLD. It should be noted that cognitive functions were negatively associated with total FFAs in LLD, especially in visuospatial/executive function. Regression analysis indicated that total FFAs negatively predicted cognitive functions. FFAs, as non-esterified fatty acids, are products of the breakdown of triglycerides, known to be lipotoxic [50] and able to permeate the brain through passive transportation or protein-mediated endocytosis, thereby affecting vascular endothelial function [51]. They were believed to forewarn the incidence of AD in patients suffering from type 2 diabetes mellitus (T2DM) [52]. Zhu [53] reported that FFAs are negatively related to cognitive function, especially attention and executive ability, in T2DM patients suffering from MCI. Holloway [54] reported that higher FFA levels were related to worse cognitive functions among healthy people. These findings revealed the effects of FFAs on cognitive functions in patients suffering from LLD. However, there were some inconsistent results. For example, studies showed that the levels of certain FFAs, such as monounsaturated fatty acids, had protective effects on cognitive function and were decreased in patients suffering from AD [55]. One probable interpretation for above contradictions was that different types of FFAs had diverse influences on cognition, the positive effects of some fatty acids may be offset by the negative effects of others. Current study measured total FFAs content.

As for gut microbiota and lipid metabolism, current results revealed that *Akkermansia* was negatively related to total FFAs. Many previous studies confirmed the correlation between *Akkermansia* and lipid metabolism [56, 57]. Zou [56] reported a correlation between *Akkermansia* and FFAs in an obese mouse model. Rodríguez-Carrio [57] reported that, in the normal population, *Akkermansia* could predict the level of FFAs in peripheral blood and was negatively correlated with this parameter, which is consistent with this study. The *Akkermansia*-host relationship was manifested in energy expenditure related to glycolipid metabolism, which affected obesity [58]. Research had shown a close inverse relationship between *Akkermansia* enrichment and obesity development [59] and T2DM [60]. This research also supplemented the literature about the correlation between *Akkermansia* and lipid metabolism in individuals with geriatric mental disorders.

Most importantly, mediation analysis, for the first time, revealed that the impact of *Akkermansia* on cognitive function in LLD was mediated by FFAs. Previous research displayed lipid metabolism level is an important factor affecting the prognosis of patients with cognitive impairment [61, 62]. Wang [61] found that cognitive decline related to altered gut microbiota among peritoneal dialysis patients, and functional analyses showed this relationship was related to fatty acid metabolism. Ou [62] found that in AD model mice transplanted with *Akkermansia*, FFAs levels decreased and cognitive function improved accordingly. *Akkermansia* existed inside the mucous intestinal layer, thereby contributing to the reinforcement of the intestinal wall [63]. Furthermore, FFAs may induce neuroinflammatory response and insulin resistance through G protein-coupled receptors overactivation, therefore causing neurotoxicity and affecting cognitive function [57]. This study was consistent with these findings and provided new explanation for the relationship between FFAs, gut microbes and cognitive function in LLD. The mediation relationship also had noteworthy implications for clinical practice. Firstly, in LLD patients, we should pay more attention to those who have dysregulation of lipid metabolism, especially elevated FFAs, as they may have a higher potential risk of cognitive deficits. Besides, regulating lipid metabolism in patients while treating depression may lead to cognitive benefits. Finally, due to the metabolic disorders caused by some antidepressants and antipsychotics, cognitive symptoms of LLD should also be concerned.

### Study strengths and limitations

This study is so far the first to investigate the relationship between cognitive function, gut microbiota and lipid metabolism in LLD patients, and emphasizes the mediating role of total FFAs in microbiome-gut-brain axis. Current study also made extensive adjustments for potential confounding factors. However, a few unignorable limitations should be taken into consideration. Firstly, the limited sample size should not be overlooked, necessitating further expansion to deepen the understanding of the pertinence of gut microbiota, lipid metabolism and cognitive functions among LLD patients. Secondly, it is a cross-sectional study that reflects the causal relation of gut microbiota, and lipid metabolism upon cognitive function in patients with LLD in a limited way.

## Conclusion

In conclusion, this study demonstrated that cognitive function was correlated with the levels of total FFAs and the relative abundance of *Akkermansia* in LLD. Furthermore, this study emphasizes the significant mediating role of total FFAs between *Akkermansia* and cognitive function, which offers new perspective into the role of gut microbial-lipid metabolism axis in the cognitive function of LLD, and could guide individualized therapeutic interventions. Clinically, the results highlight the importance of fluctuation of serum lipid metabolism markers, especially total FFAs, which may imply the gut microbiota dysregulation and further potential cognitive impairment in LLD. Furthermore, lipid metabolism management may have clinical benefits for cognitive function in LLD. For the future perspectives, further revealing the precise interactions between gut microbiota and lipid metabolism may provide mechanistic insights and help to develop new therapeutic approaches for cognitive impairment in LLD.

## Data Availability

The data supporting the results of our study are stored in the OMIX repository: https://download.cncb.ac.cn/OMIX/OMIX004832/.

## Abbreviations

LLD: Late-life depression
MoCA: Montreal Cognitive Assessment
FFAs: Free Fatty Acids
AD: Alzheimer’s disease
ApoE: Apolipoprotein E(ApoE)
ApoC1: Apolipoprotein C1
CLU: Clusterin
BMI: Body-mass index
MDD: Major Depressive Disorder
DSM-V: The Diagnostic and Statistical Manual of Mental Disorders, Fifth Edition
GDS-30: 30-Item Geriatric Depression Scale
HDL: High density lipoprotein
LDL: Low density lipoprotein
ApoA1: Apolipoprotein A1(ApoA1)
ApoB: Apolipoprotein B(ApoB)
TC: Total cholesterol
TG: Triglyceride
LPPA: Lipoprotein A
OTU: Operational taxonomic unit
SD: Standard deviation
CI: Confidence interval
MCI: Mild Cognitive Impairment
T2DM: Type 2 diabetes mellitus
